# Patients with chronic post‐dural puncture headache do not have typical imaging features of intracranial hypotension: An MRI study using the Bern score

**DOI:** 10.1111/head.15057

**Published:** 2025-09-23

**Authors:** Charlotte Zander, Christian Fung, Amir El Rahal, Florian Volz, Katharina Wolf, Alexander Rau, Hansjörg Mast, Jürgen Beck, Horst Urbach, Niklas Lützen

**Affiliations:** ^1^ Department of Neuroradiology, Medical Center – University of Freiburg, Faculty of Medicine University of Freiburg Freiburg Germany; ^2^ Department of Neurosurgery Lindenhofspital of Bern Bern Switzerland; ^3^ Department of Neurosurgery, Medical Center – University of Freiburg, Faculty of Medicine University of Freiburg Freiburg Germany

**Keywords:** cerebrospinal fluid, diagnostic imaging, intracranial hypotension, lumbar puncture, orthostatic headache, post‐dural puncture headache

## Abstract

**Objective:**

This study evaluated cranial magnetic resonance imaging (MRI) signs in patients with post‐dural puncture headache (PDPH) using an established assessment score developed for spontaneous intracranial hypotension (Bern score). We hypothesize that patients with chronic PDPH do not have typical imaging features of intracranial hypotension.

**Background:**

PDPH is a well‐known complication following an intentional or unintentional lumbar dural puncture with positional headache, neck stiffness, and hearing disturbances usually resolving within 14 days. However, the chronic course of PDPH is poorly represented in the third version of the International Classification of Headache Disorders (ICHD‐3). Moreover, data on the role of cranial MRI in this cohort are lacking, but could facilitate care and management of chronic PDPH.

**Methods:**

In this post hoc retrospective case series based on a chart review, we identified 86 consecutive patients from a tertiary medical care center in Freiburg, Germany between 01/2018 and 10/2024 with chronic PDPH, defined as persisting symptoms for >14 days post puncture and/or persisting after one or more epidural blood patches (EBP). Inclusion criteria were history of lumbar puncture (LP) or unintended dural puncture (UDP) and contrast enhanced cranial MRI for assessment of Bern score in all patients. Presence of epidural lumbar fluid was evaluated using heavily T2‐weighted MRI or computed tomography (CT) myelography, whenever available (83/86 patients). Data were reviewed independently and blinded by two radiologists.

**Results:**

Eighty‐six patients with chronic PDPH (66 females; mean age of 38.8 ± 11.2 SD years) were included with LP as primary cause in 72% (*n*=62) and UDP while peridural (synonymous epidural) anesthesia (PDA) in 28% (*n* = 24). Median symptom duration was 220.0 (interquartile range [IQR] 94.0‐474.0) days. Overall median Bern score was 2.0 (IQR 1.0‐3.0) with no significant differences between LP versus PDA (*p* = 0.379). Local epidural fluid was present in 9/83 (11%) cases with adequate imaging and accompanied by higher median Bern scores (5.0 vs. 2.0; *p* = 0.026). Prior EBP was linked to lower median Bern scores (1.0 vs. 3.5; *p* < 0.001).

**Conclusion:**

Patients with chronic PDPH predominantly present a low Bern score and rarely exhibit spinal epidural fluid. If present, spinal epidural fluid is accompanied by higher Bern score. Our findings highlight the unreliability of current MRI diagnostics to detect patients with chronic PDPH, which must not lead to a mitigation of the diagnosis or a refusal of treatment. Further research on MRI markers is needed here.

## Plain Language Summary

Post‐dural puncture headache (PDPH) is a common complication after lumbar puncture or unintended dural puncture and may become chronic. In this study, we used cranial MRI to evaluate the Bern score in a group of patients with chronic PDPH. Our results suggest that current MRI workups are not helpful in detecting this type of headache disorder and further research on diagnostic imaging markers in this cohort is needed; additionally, clinicians should be aware that a normal MRI does not rule out the diagnosis of chronic PDPH.

**Abbreviations**
cPDPH: chronic post‐dural puncture headache
CSF: cerebrospinal fluid
CT: computed tomography
CTM: CT myelography
EBP: epidural blood patch
ICHD‐3: 3rd Version of the International Classification of Headache Disorders
IQR: interquartile range
LP: lumbar puncture
MRI: magnetic resonance imaging
PDA: peridural anesthesia
PDPH: post‐dural puncture headache
SIH: spontaneous intracranial hypotension
UDP: unintentional dural puncture

## INTRODUCTION

According to the third version of the International Classification of Headache Disorders (ICHD‐3), post‐dural puncture headache (PDPH) is defined as headache occurring within 5 days after a lumbar puncture due to leakage of cerebrospinal fluid (CSF) through the dural defect, often accompanied by neck stiffness and/or subjective hearing symptoms.^1^ The dural defect can either be caused by an intentional lumbar puncture (LP) for diagnostic or therapeutic reasons, or an unintentional dural puncture (UDP), e.g. in context of peridural procedures, such as peridural anesthesia (PDA), synonymous with epidural anesthesia (EDA).^2^ Several risk factors are known to cause PDPH, including the type of needle and the position of the bevel in relation to the longitudinal fibers of the dura as well as patient's position during lumbar puncture.^3^, ^4^ The reported incidence of PDPH varies between 2% and 40% and can lead to serious complications such as subdural hematomas.^2^, ^5^, ^6^

The long‐term harms of PDPH are barely considered in the current ICHD‐3 criteria, suggesting self‐limitation of the disease within two weeks or after epidural blood patch (EBP).^1^ Recent studies indicate that PDPH can cause chronic head and neck pain that can last even for years with substantial impact on quality of life.^7^, ^8^, ^9^, ^10^, ^11^ This emphasizes the need for a proper diagnostic workup and assessment of this disorder.

Magnetic resonance imaging (MRI) may facilitate diagnosing patients with PDPH using objective parameters, although these have not yet been sufficiently investigated.^2^ While pachymeningeal enhancement is known in acute PDPH, imaging signs in patients with chronic PDPH (cPDPH) are not established,^12^ and MRI was recently reported to be unremarkable in this cohort.^13^ This might lead to missed diagnoses with potentially delaying or even denying treatment in these patients, which in turn increases the risk of chronification.^7^, ^14^

To evaluate the presence of MRI alterations in cPDPH, we applied the Bern score, which has become established to calculate the likelihood of a spinal CSF leak using contrast‐enhanced cranial MRI.^15^ Initially, it was designed for patients with spontaneous intracranial hypotension (SIH) with ventral and lateral dural tears (type 1 and 2 leaks) and later validated for patients with CSF‐venous fistula (type 3).^15^, ^16^ SIH and PDPH are both believed to be caused by a loss of cerebrospinal fluid. Therefore, one might expect similar signs to be found in cranial MRI.

We hypothesize that patients with chronic PDPH do not have typical imaging features of intracranial hypotension. In this study, we investigated the presence of imaging features indicative of dural leakage in a cPDPH cohort, potentially contributing to a better understanding and management of this largely unknown disease.

## MATERIALS AND METHODS

### Study design

This is a retrospective case series based on a chart review with a post hoc primary analysis of all collected data. The study was approved by the local Institutional Review Board (24‐1296‐S1‐retro). All patients provided informed written consent.

### Study population

We retrospectively identified all patients with PDPH according to the ICHD‐3 criteria^1^ admitted to our tertiary medical care center between January 2018 and October 2024 who had received contrast‐enhanced cranial MRI in our department. We defined cPDPH as a history of intended or unintended dural puncture with subsequent symptoms suggestive of CSF loss, either persisting >14 days post‐puncture and/or persisting after one or more EBPs.

Patients with a symptom duration of less than 15 days i.e. acute PDPH or postoperative CSF leakage, the presence of hydrocephalus, and suspected meningeal carcinomatosis were excluded.

### Variables assessed

Demographic data were extracted from the patients' charts and included sex, age, and body mass index (BMI). Time data for the dural puncture were taken from the patients' medical history. Where available, the exact date of symptom onset was used to calculate symptom duration. In some cases, however, only the month of LP/UDP was specified; in these cases, the 15th of each month was used uniformly for the calculation. In addition, the cause for the lumbar puncture was specified (LP due to diagnostic or therapeutic reasons or UDP due to peridural or epidural procedures). It was also recorded whether the patients had already received one or more EBPs before the MRI scan. To investigate potential differences in MRI findings, we conducted a group comparison between patients with cPDPH after UDP during PDA (PDA subgroup) versus LP (LP subgroup).

### Imaging specifications and evaluation

Consistent with the diagnostic workup in SIH,^17^ our institutional standard for MRI examination in PDPH does not only cover cranial, but also lumbar spinal imaging to search for epidural fluid collections, which prove a dural tear.^18^ For this study, cranial and spinal imaging was assessed independently by two radiologists (N.L., C.Z.), blinded to clinical data and prior imaging results. In cases of disagreement, the decision was made by consensus.

MRI was performed on a 1.5 or 3 T scanner (Magnetom Avanto, Magnetom Prisma; Siemens Healthineers, Erlangen, Germany) using standard head–neck‐spine 12, 4, 24 channel (1.5 T) and 44, 20, 32 channel (3 T) coils.

The first inhouse contrast‐enhanced cranial MRI was used to calculate the Bern score^15^ including the assessment of presence of pachymeningeal enhancement (2 points), enlargement of venous sinus (2 points), suprasellar distance <4.0 mm (2 points), mamillopontine distance <6.5 mm (1 point), prepontine distance <5.0 mm (1 point), and presence of subdural fluid (1 point).^15^

Presence of local epidural fluid collections in the lumbar spine was assessed using fat‐saturated, heavily T2‐weighted 3D sequences (T2w Spectral Attenuated Inversion Recovery; SPAIR or T2w Sampling Perfection with Application optimized Contrast using different flip angle Evolution; SPACE fat‐sat) or results of invasive computed tomography (CT) myelography (CTM) if spinal CT imaging was carried out within 14 days around cranial MRI.

MRI sequence parameters of the head and spine are summarized in Table S1.

Before the introduction of 3D heavily T2‐weighted spinal MRI at our institute in November 2021, presence of epidural fluid at the spine was investigated with conventional invasive CTM if deemed necessary. Following a lumbar puncture, 15 mL of iodine contrast agent (250 mg iodine/mL; Iopamidol 250M, Bracco, Germany) were injected and CTM in the supine position was performed with a delay of 10–20 min. As studies demonstrated no superiority of the conventional CTM compared to heavily T2‐weighted spinal MRI for the detection of epidural fluid (indicating a dural tear), our institutional standard changed accordingly.^19^ Therefore, if no adequate heavily T2‐weighted MRI of the spine was available, conventional CTM was used to assess epidural fluid/contrast agent where present. Particular care was required to distinguish an extradural contrast medium release from a pre‐existing dural tear and the current iatrogenic puncture site during myelography (which is usually less longitudinally aligned); in cases of doubt, this was clarified by consensus.

### Statistical analysis

No statistical power calculation was conducted prior to the study. The sample size was based on the available data. The assumption of normal distribution was tested with the Shapiro–Wilk test. Descriptive analysis was performed using frequencies and percentages for categorical variables and mean (±SD) or median (interquartile ranges [IQRs]) for continuous and ordinal variables. Group analyses were assessed with independent samples *t*‐tests, Mann–Whitney *U* test, and chi‐squared test as appropriate. A potential association of Bern score and symptom duration was investigated using Spearman's rank correlation coefficient. All hypothesis tests were two‐tailed and considered significant at an alpha level of 0.05. Statistical analyses were performed using jamovi version 2.5 (The jamovi project, Sydney, Australia) and R version 4.3.0 (R Foundation for Statistical Computing, Vienna, Austria).

## RESULTS

### Patient characteristics

**FIGURE 1:**
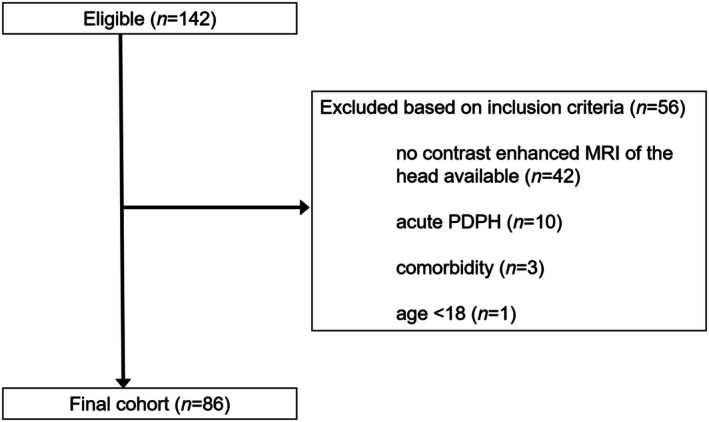
Flow chart of the study population. Between January 2018 and October 2024, a total of 142 patients were retrospectively identified who were admitted to our tertiary medical care center and met ICHD‐3 criteria for PDPH. After applying the exclusion criteria, 86 patients were included in the final analysis. ICHD‐3, third Version of the International Classification of Headache Disorders; MRI, magnetic resonance imaging; PDPH, post‐dural puncture headache.

Between January 2018 and October 2024, a total of 142 patients were retrospectively identified who were admitted to our tertiary medical care center and met ICHD‐3 criteria for PDPH.^1^ Of these, 42 were excluded because no contrast‐enhanced cranial MRI was performed at our institution. Additionally, one patient under the age of 18 years and three patients with comorbidities that could impede the assessment of the Bern score were excluded. From the remaining 96 patients, 10 presented with acute PDPH (symptom duration ≤14 days), resulting in 86 patients being included in the final cohort for analysis (see Figure 1). Except for missing lumbar imaging in 3/86 patients, there were no missing data.

Patients' demographic and clinical data are summarized in Table 1. Of 86 patients with cPDPH, 66 (77%) were female, mean age was 38.8 years (±11.2, range 18–77), and mean BMI was 24.3 (±4.7, range 14.9–37.8). Diagnostic LP was identified as the primary cause of cPDPH in 55 patients (64%) with headache as cause for the LP in 14/55 cases (26%). Therapeutic LP for therapy of idiopathic intracranial hypertension was causative in two patients (2%), while UDP due to periradicular therapy was causative for five patients (6%). UDP during PDA accounted for 21 cases (24%), two patients had PDPH after lumbar drainage (2%), and one patient developed symptoms after implantation of a spinal cord stimulator (1%).

**TABLE 1:** Patients’ characteristics of the overall cohort with chronic postdural puncture headache and in comparison between the subgroup after LP versus PDA.

Variable	All (*n* = 86)	LP (*n* = 62)	PDA (*n* = 24)	LP versus PDA
Age, years (mean [SD])	38.8 [11.2]	38.9 [10.8]	38.4 [12.5]	*p* = 0.640
Female (%) Male (%)	66 (77%) 20 (23%)	44 (71%) 18 (29%)	22 (92%) 2 (8%)	*p* = 0.042
Body mass index, kg/m^2^ (mean [SD])	24.3 [4.7]	24.3 [5.1]	24.2 [3.52]	*p* = 0.685
Symptom duration, days (median [IQR])	220.0 [94.0–474.0]	188.0 [82.5–400.0]	360.0 [158.0–879.0]	*p* = 0.046
Bern Score (median [IQR])	2.0 [1.0–3.0]	2.0 [1.0–3.0]	2.0 [1.0–4.0]	*p* = 0.379
Epidural fluid (%)				
Yes	9 (11%)	5 (8%)	4 (17%)	*p* = 0.276
No	74 (89%)	54 (92%)	20 (83%)	
Prior blood patch (%)				
Yes	62 (72%)	45 (73%)	17 (71%)	*p* = 0.871
No	24 (28%)	17 (27%)	7 (29%)	

*Note*: Data are reported as mean (SD) or median (IQR) where appropriate. Numeric variables were tested with independent *t*‐test or Mann–Whitney‐*U* test, whereas nominal variables were tested using chi‐squared test.

Abbreviations: IQR, interquartile range; LP, lumbar puncture; PDA, peridural anesthesia; SD, standard deviation.

Median symptom duration was 220.0 (IQR 94.0–474.0, range 15–4050) days. Symptom duration was significantly longer in the PDA subgroup versus LP subgroup (median 360.0 days vs. median 188.0 days; *p* = 0.046). Notably, one case after PDA had a very long duration with more than 11 years of symptoms. We did not find a significant association between the Bern score and symptom duration (Spearman's rho = −0.069, *p* = 0.265).

A total of 62 patients (72%) did receive one or more EBPs before the initial MRI in our department (median 1.0, IQR 0–2.75, range 0–7). Furthermore, four patients already had surgical exploration for closure of the leak.

### Imaging findings of the head

The overall median Bern score in the entire cohort was 2.0 (IQR 1.0–3.0, range 0–9), and the median Bern score did not differ significantly between LP versus PDA subgroup (2.0 vs. 2.0, *p* = 0.379). We thus found a low likelihood for spinal CSF leak as defined by Dobrocky et al.^15^ for 54 patients (63%), an intermediate probability for 26 patients (30%), and high probability for six (7%).

**FIGURE 2:**
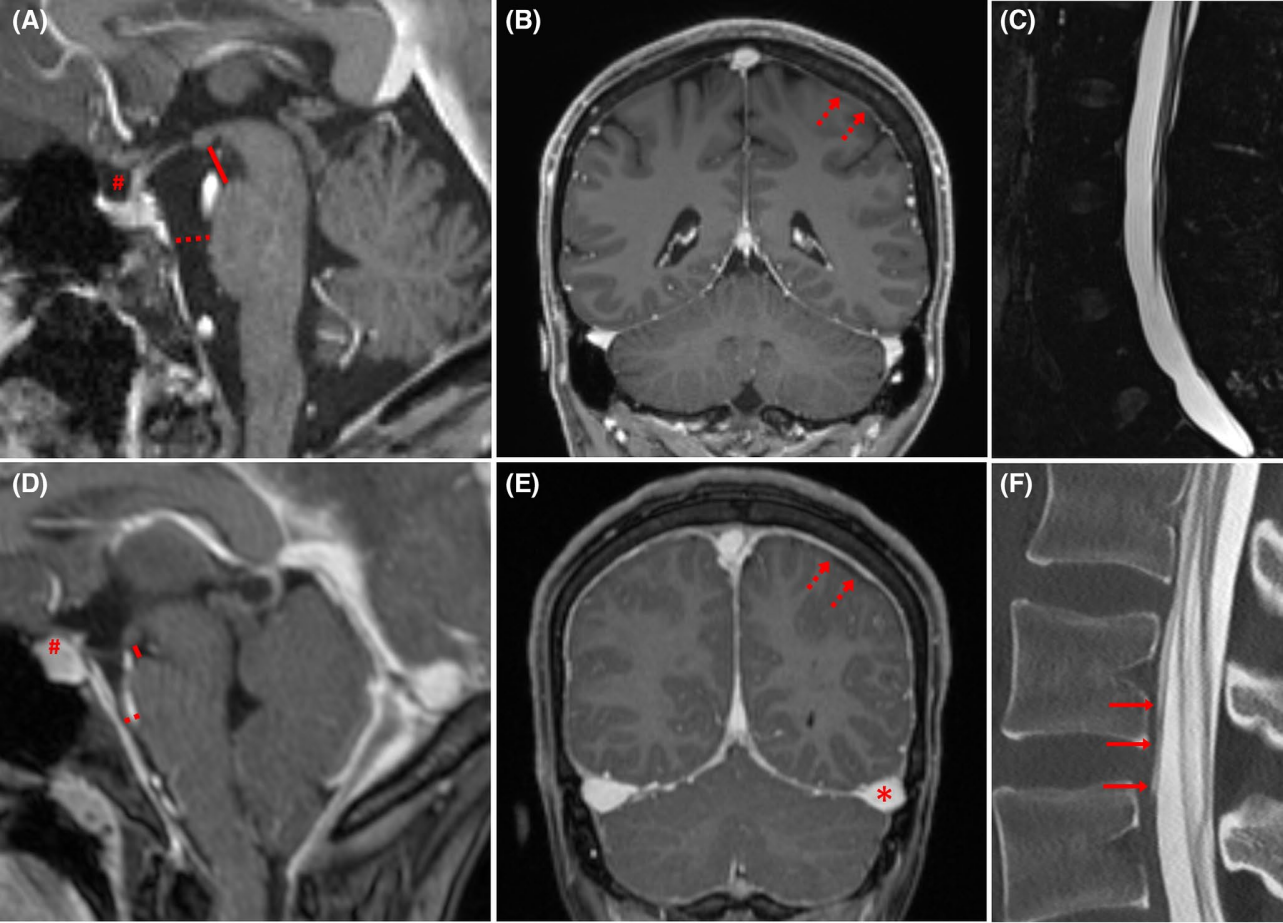
Imaging in two patients with chronic post‐dural puncture headache after unintentional dural puncture show representative examples of both, unremarkable (A‐C) and positive (D‐E) findings for spinal CSF loss. Contrast‐enhanced sagittal (A) and coronal (B) T1‐weighted MPRAGE in a 31‐year‐old woman shows normal measurements of the suprasellar (rhomb), prepontine (red dotted line), and mamillopontine (red line) distances in (A) as well as unenhanced dura (dotted arrow in B). Sagittal 3D T2‐weighted SPACE fs at a spatial resolution of 0.63 mm is negative for epidural lumbar fluid (C). Contrast‐enhanced sagittal (D) and coronal (E) T1‐weighted MPRAGE in a 27‐year‐old woman discloses positive findings of the head for a spinal CSF leak including diminished mamillopontine, prepontine, and suprasellar distances as well as engorgement of the venous sinus (asterisk) and pachymeningeal enhancement (dotted arrow) with small subdural hematoma/hygroma (D). Moreover, contrast egress is visible in the sagittal CT myelography after intrathecal injection of iodine contrast medium in the ventral epidural space at the level L1/2 (arrows in F). CSF, cerebrospinal fluid; CT, computed tomography; fs, fat‐sat; MPRAGE, 3D Magnetization Prepared Rapid Gradient Echo; SPACE, 3D Single slab turbo spin echo (TSE) sequence with a slab selective, variable excitation pulse. [Color figure can be viewed at wileyonlinelibrary.com]

A reduced prepontine distance was found in 50/86 patients (58%), which therefore was the most common subitem in our cohort. Diminished values of the mamillopontine distance were found in 46/86 patients (54%) and of the suprasellar cistern in 29/86 patients (34%). Pachymeningeal enhancement was rated positive in 13/86 cases (15%) while engorgement of the venous sinus and subdural fluid were present in 5/86 (6%) patients, respectively. Exemplary cases are shown in Figure 2.

**FIGURE 3:**
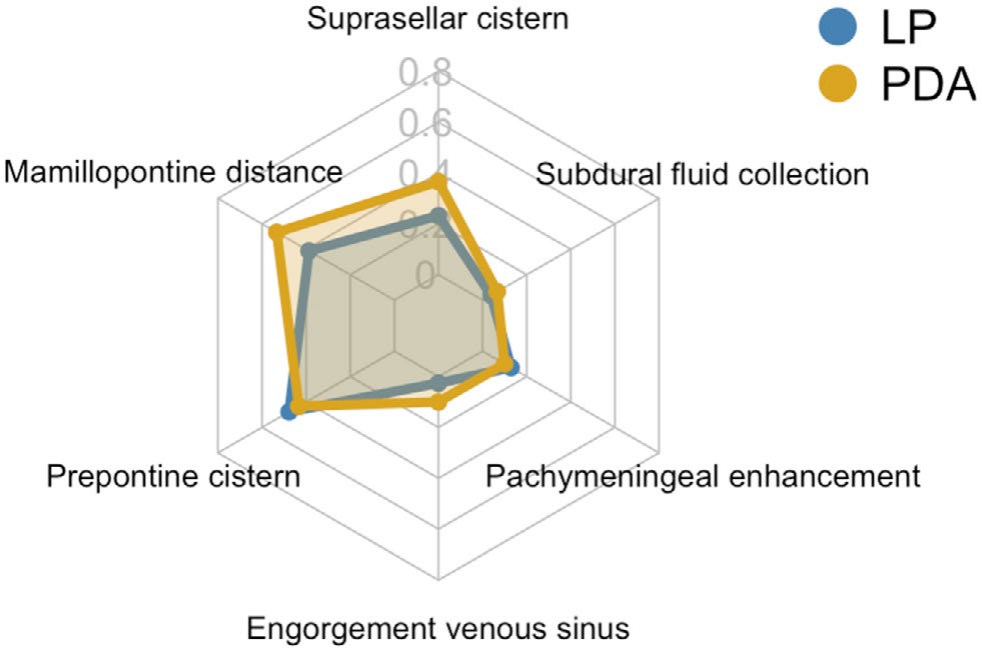
Radar chart for visualization of the distribution of the different Bern score criteria in our cohort of 86 patients with chronic post‐dural puncture headache in the lumbar puncture (LP) versus peridural anesthesia (PDA) group. The scale ranges from 0 to 0.8, with 0.2 increments, representing the frequency of each criterion in the respective groups. In all patients with chronic post‐dural puncture headache due to LP, reduction of the prepontine cistern as well as reduced mamillopontine distance was the most frequent sign in the Bern score. A similar pattern was found in the group after PDA with reduction of mamillopontine distance being the most frequent sign. Major criteria of the Bern score were rare in our cohort. [Color figure can be viewed at wileyonlinelibrary.com]

**FIGURE 4:**
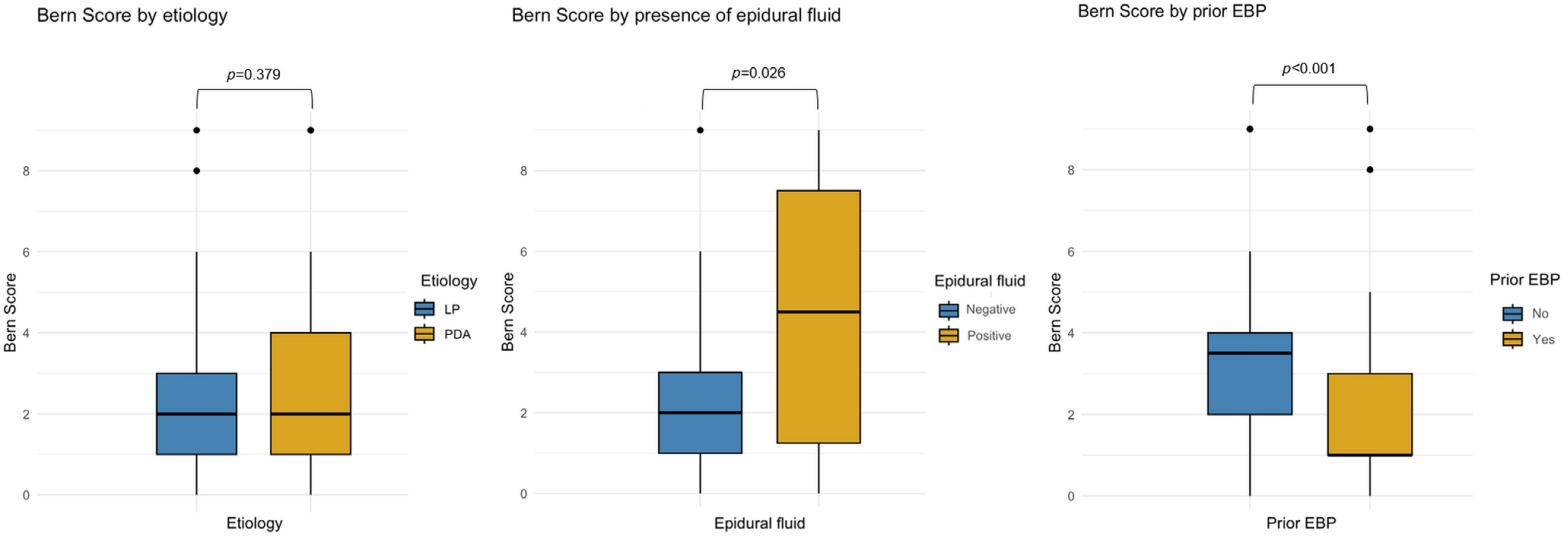
Determinants of the Bern score in 86 patients with chronic post‐dural puncture headache. Median Bern score shows comparable values in the lumbar puncture (LP) cohort versus peridural anesthesia (PDA) cohort. Positive finding of lumbar epidural fluid was accompanied with higher Bern scores. Conversely, prior epidural blood patch (EBP) led to lower scores. [Color figure can be viewed at wileyonlinelibrary.com]

We found no significant differences in the presence of individual Bern score features between the LP and PDA subgroups (suprasellar cistern *p* = 0.139, mamillopontine distance *p* = 0.127, prepontine distance *p* = 0.642, pachymeningeal enhancement *p* = 0.673, engorgement of venous sinus *p* = 0.099, subdural fluid *p* = 0.534). The distribution of the individual minor and major features is shown in Figure 3 and Table S2. Prior EBP was linked to lower median Bern score (1.0 vs. 3.5, *p* < 0.001; see Figure 4).

### Imaging findings of the lumbar spine

MRI was available in 69/83 cases (83%) and invasive conventional CTM in 14/83 patients (17%). Local epidural fluid was found in 5/59 (8%) patients after LP and 4/24 (20%) patients after PDA. Presence of lumbar epidural fluid was accompanied by significantly higher median Bern scores (5.0 vs. 2.0, *p* = 0.026). There was a tendency towards shorter symptom duration in the subgroup positive for epidural fluid versus patients negative for epidural fluid, however this tendency did not reach significance (116.0 days vs. 245.0 days, *p* = 0.102).

## DISCUSSION

PDPH constitutes a serious complication following intentional or unintentional dural puncture and can develop into a chronic condition.^7^ Our systematic evaluation of imaging signs in a large cohort of cPDPH with long standing symptoms revealed rather low Bern scores, indicating a low probability for finding a spinal CSF leak, and epidural fluid was found in only 11%, which in turn was linked to higher Bern scores.

Recent studies suggest that cPDPH is more prevalent than previously thought.^7^, ^9^, ^11^, ^20^ The current ICHD‐3 guidelines define PDPH as headache occurring within 5 days after LP, resolving spontaneously within 14 days or after sealing the leak with EBP. Persistent symptoms continuing beyond 14 days are, however, not acknowledged.^1^ In 2021, the American Society of Anesthesiologists proposed that the diagnosis of PDPH should be made only in view of clinical presentation, detailed history, and physical examination.^21^ In the consensus practice guidelines of 2023, MRI in PDPH is only recommended if atypical symptoms or neurological deficits are present, whereby data available at that time were classified as insufficient to assess the role of routine imaging.^2^ However, the reality often deviates from this: in clinical practice, imaging is often used, on the one hand, driven by uncertainty, as cPDPH in particular is hardly known, and, on the other hand, by the expectation of positive MRI findings similar to patients with SIH, as characterized by the established Bern score and spinal epidural fluid. Therefore, the evaluation of imaging data in these patients is essential to understand both the acute and chronic conditions of PDPH.

Our study reveals that patients with cPDPH do not have typical MRI signs usually expected in patients with intracranial hypotension such as SIH (or acute PDPH).^12^, ^15^, ^22^ The median Bern score in our cohort was 2.0 corresponding to a low probability for intracranial hypotension, with no differences between the LP and PDA (synonymous EDA) subgroup.^15^ These results are consistent with previous studies indicating predominantly normal cranial MRI scans in patients with cPDPH in smaller cohorts.^14^, ^18^, ^23^

In the original study from Dobrocky et al. to determine the Bern score, the most common finding in patients with SIH was pachymeningeal enhancement in 77% and venous engorgement in 66% of cases, specified as major criteria.^15^ In contrast, pachymeningeal enhancement was present in only 13/86 (15%) and venous engorgement in 5/86 (6%) of patients in our cohort, whereas minor criteria were more common with a reduced prepontine distance in 50/86 patients (58%) and diminished values of the mamillopontine distance in 46/86 patients (54%). A recent study evaluating the Bern score in healthy controls also found diminished mamillopontine and prepontine distance in a relevant rate, suggesting that presence of these signs alone might not be sufficient for diagnosis of CSF loss.^24^

In their original control group (*n* = 60), a Bern score ≤2 points (low probability for finding a spinal CSF leak) ruled out SIH with a sensitivity and a specificity of 93% each,^15^ whereas 63% diagnosed with cPDPH in our cohort had a Bern score ≤2 points. Considering a high Bern score of ≥5 (high probability for finding a spinal CSF leak), the original study enabled detection of SIH with a sensitivity of 79% and specificity of 98%,^15^ whereas 93% of the cPDPH patients in our cohort would have been missed at this cutoff value.

In contrast, studies on patients with acute PDPH (≤14 days symptom duration) commonly show positive signs for CSF loss on cranial MRI,^12^, ^22^ in particular pachymeningeal enhancement.^12^ The minor proportion of pachymeningeal enhancement in our cohort could be related to the long median symptom duration, as it is known that imaging signs of the head (especially pachymeningeal enhancement) decrease over time in SIH.^25^ However, we found no significant correlation between disease duration and the Bern score, but there was a tendency towards shorter symptom duration in patients with positive findings of epidural fluid. The non‐significant testing may be the result of one outlier with a symptom duration of 4064 days after PDA who was still positive for epidural fluid at the time of the spinal MRI.

The presence of a spinal epidural fluid collection proves a dural tear. In our cohort, only a minority of patients showed local epidural fluid (9/83 patients with adequate imaging, 11%). To date, there are only few investigations addressing this topic. One study by Schievink et al. reported similar results with 19% showing epidural fluid in a cohort of 27 patients with cPDPH.^18^ Of note, the presence of local epidural fluid was related to a significantly higher median Bern score in our cohort. Studies have already demonstrated that the type of needle (which differs depending on the intervention) influences the development of PDPH.^26^, ^27^ Since UDP after PDA is associated with larger and typically cutting needles (usually 16–18G with PDA vs. 20–25G with LP),^4^, ^26^, ^27^, ^28^ we have expected a higher proportion of epidural fluid in the PDA subgroup, which we did not observe in our cohort.

The main finding of this study is the low prevalence of MRI signs of intracranial hypotension, as measured by the Bern score, in our cohort of patients with cPDPH, which is contrary to the tremendous impact on the patients' socioeconomic life.^3^, ^7^, ^8^, ^9^, ^11^, ^13^ These results are counterintuitive and show that the chronicity of this disease is not understood yet and requires further research (including imaging). Moreover, cPDPH is usually not accompanied by spinal epidural fluid.

There are other imaging signs, such as a reduced pontomesencephalic angle,^29^ the venous hinge angle,^30^ and layered calvarial hyperostosis,^31^ but also the recently described perioptic CSF space.^32^ These features were not specifically assessed in this paper, but may be of interest for future studies. Two further imaging signs with uncertain significance have been reported: the “dinosaur tail sign” can be visible on heavily T2‐weighted spinal MRI and is discussed as a possible correlate of interspinous fluid collection, but has only been investigated in patients with acute PDPH.^33^ Others reported an “arachnoid bleb” on MRI or CTM which may indicate a small arachnoid layer herniating through the lumbar dural tear that was caused by an intentional or unintentional puncture.^34^, ^35^ However, these signs can be subtle and easily overlooked. Further imaging signs may help to understand and monitor the disease in order to improve patients' outcome. Guidelines revised in the future may include the chronic condition of PDPH.

## LIMITATIONS

Our study has several limitations: First, the retrospective, monocentric single‐armed design introduces inherent limitations, such as the inability to infer causality and the risk of selection bias regarding both patient inclusion and treatment approaches. Although we attempted to minimize selection bias by including all consecutive patients who fulfilled the inclusion criteria, the single‐center data collection likely resulted in a cohort representing particularly severe, complex, and treatment‐resistant cases of cPDPH, which may not be representative of the broader cPDPH population.

Second, some of the patients already received one or more EBP and/or even surgical exploration before cranial MRI, which could influence imaging signs, potentially confounding the interpretation of MRI signs. Nevertheless, all patients collected for this study were still symptomatic for PDPH.

Third, MRI scans were performed at widely varying time points relative to symptom onset, introducing significant heterogeneity. This variability may obscure time‐dependent imaging findings and limits the ability to draw uniform conclusions across the entire cohort.

Fourth, since the PDA subgroup with 24 patients is relatively small, the subgroups after LP versus PDA are not balanced limiting comparability. This increases the risk of type II errors and restricts the comparability between subgroups.

However, to our knowledge, this is the largest cohort to date to investigate this specific entity.

## CONCLUSION

This study indicates that patients with cPDPH, defined as persisting symptoms for >14 days after intended or unintended dural puncture and/or persisting after one or more EBP, largely escape detection by diagnostic imaging. On cranial MRI they predominantly presented a low Bern score, usually indicating a low probability of finding a spinal CSF leak and spinal epidural fluid is mostly absent in these patients. However, this must not lead to mitigation of diagnosis or refusal of treatment. As chronic courses of PDPH are increasingly recognized and not yet fully understood, further research is needed here.

## AUTHOR CONTRIBUTIONS

**Charlotte Zander:** Writing – original draft; writing – review and editing; visualization; validation; investigation; formal analysis; data curation; resources; conceptualization; methodology. **Christian Fung:** Conceptualization; methodology; writing – review and editing. **Amir El Rahal:** Writing – review and editing; resources. **Florian Volz:** Writing – review and editing; resources. **Katharina Wolf:** Resources; writing – review and editing. **Alexander Rau:** Writing – review and editing; formal analysis; data curation; visualization. **Hansjörg Mast:** Resources; writing – review and editing. **Jürgen Beck:** Writing – review and editing; resources; project administration. **Horst Urbach:** Resources; writing – review and editing; project administration. **Niklas Lützen:** Supervision; resources; project administration; conceptualization; investigation; writing – original draft; writing – review and editing; methodology; validation; data curation.

## FUNDING INFORMATION

None.

## CONFLICT OF INTEREST STATEMENT

**Charlotte Zander, Christian Fung, Amir El Rahal, Florian Volz, Katharina Wolf, Alexander Rau, Hansjörg Mast, Jürgen Beck, Horst Urbach**, and **Niklas Lützen** have no competing financial and/or non‐financial interests in relation to the work described.

## TRIAL REGISTRATION

DRKS00034978.

## Supporting information


**Table S1:** Technical data of used imaging protocols for head and spine for Prisma (3 T)/Avanto (1.5 T). All patients included in the study received 3D T1‐weighted contrast enhanced magnetic resonance imaging of the brain (MPRage). For assessment of epidural lumbar fluid, heavily‐T2‐weighted imaging of the lumbar spine was used, where available (i.e. 3D T2‐weighted SPAIR or 3D T2‐weighted SPACE fat‐sat). Imaging was either done in 1.5 Tesla scanner (Avanto, Siemens, Erlangen, Germany) or 3 Tesla (Prisma, Siemens, Erlangen, Germany). FA, flip angle; MPRAGE, 3D Magnetization Prepared Rapid Gradient Echo; SPACE, sampling perfection with application‐optimized contrasts by using different flip angle evolutions; SPAIR, spectral attenuated inversion recovery; TE, echo time; TR, repetition time.
**Table S2:** Results of the evaluation of the Bern score in 86 chronic postdural puncture headache patients comparing each minor and major criterium between the subgroups after lumbar puncture (LP) versus after peridural anesthesia (PDA). After testing using the chi‐squared test, no significant differences were found for any of the signs between the two subgroups.

## References

[1] Headache Classification Committee of the International Headache Society (IHS) . The International Classification of Headache Disorders, 3rd edition. Cephalalgia. 2018;38(1):1‐211. 10.1177/0333102417738202 29368949

[2] Uppal V , Russell R , Sondekoppam R , et al. Consensus practice guidelines on postdural puncture headache from a multisociety, international working group: a summary report. JAMA Netw Open. 2023;6(8):e2325387. 37581893 10.1001/jamanetworkopen.2023.25387

[3] Weji BG , Obsa MS , Melese KG , Azeze GA . Incidence and risk factors of postdural puncture headache: prospective cohort study design. Perioper Med. 2020;9(1):32. 10.1186/s13741-020-00164-2 PMC7650180 33292510

[4] Nath S , Koziarz A , Badhiwala JH , et al. Atraumatic versus conventional lumbar puncture needles: a systematic review and meta‐analysis. Lancet. 2018;391(10126):1197‐1204. 29223694 10.1016/S0140-6736(17)32451-0

[5] Sachs A , Smiley R . Post‐dural puncture headache: the worst common complication in obstetric anesthesia. Semin Perinatol. 2014;38(6):386‐394. 25146108 10.1053/j.semperi.2014.07.007

[6] Whiteley SM , Murphy PG , Kirollos RW , Swindells SR . Headache after dural puncture. BMJ. 1993;306(6882):917‐918. 8490423 10.1136/bmj.306.6882.917 PMC1677349

[7] Kraus LM , Häni L , El Rahal A , et al. Chronic post‐dural puncture headache–a serious and underrated complication following lumbar puncture: a cohort study. Front Neurol. 2024;15:1493303. doi:10.3389/fneur.2024.1493303 39677860 PMC11638535

[8] Kapan A , Waldhör T , Schiffler T , Beck J , Wöber C . Health‐related quality of life, work ability and disability among individuals with persistent post‐dural puncture headache. J Headache Pain. 2024;25(1):64. 38658862 10.1186/s10194-024-01765-8 PMC11040840

[9] Ranganathan P , Golfeiz C , Phelps AL , et al. Chronic headache and backache are long‐term sequelae of unintentional dural puncture in the obstetric population. J Clin Anesth. 2015;27(3):201‐206. 25483233 10.1016/j.jclinane.2014.07.008

[10] Webb CAJ , Weyker PD , Zhang L , et al. Unintentional dural puncture with a Tuohy needle increases risk of chronic headache. Anesth Analg. 2012;115(1):124‐132. 22467897 10.1213/ANE.0b013e3182501c06

[11] Ansari JR , Barad M , Shafer S , Flood P . Chronic disabling postpartum headache after unintentional dural puncture during epidural anaesthesia: a prospective cohort study. Br J Anaesth. 2021;127(4):600‐607. 34548152 10.1016/j.bja.2021.05.020

[12] Sánchez García FJ , Jornet Fayos J , Pastor Del Campo A , LLopis Calatayud JE . Brain MRI features of postdural puncture headache. Reg Anesth Pain Med. 2025;50(4):352‐357. doi:10.1136/rapm-2023-105105 38388010

[13] Niraj G , Mushambi M , Gauthama P , et al. Persistent headache and low back pain after accidental dural puncture in the obstetric population: a prospective, observational, multicentre cohort study. Anaesthesia. 2021;76(8):1068‐1076. 33891312 10.1111/anae.15491

[14] Kapan A , Waldhör T , Schiffler T , Beck J , Wöber C . Diagnostic and therapeutic insights in individuals with persistent post‐dural puncture headache: a cross‐sectional study. Headache. 2024;64(8):1015‐1026. 39012072 10.1111/head.14790

[15] Dobrocky T , Grunder L , Breiding PS , et al. Assessing spinal cerebrospinal fluid leaks in spontaneous intracranial hypotension with a scoring system based on brain magnetic resonance imaging findings. JAMA Neurol. 2019;76(5):580‐587. 30776059 10.1001/jamaneurol.2018.4921 PMC6515981

[16] Kim DK , Carr CM , Benson JC , et al. Diagnostic yield of lateral decubitus digital subtraction myelogram stratified by brain MRI findings. Neurology. 2021;96(9):e1312‐e1318. 33472917 10.1212/WNL.0000000000011522

[17] Cheema S , Anderson J , Angus‐Leppan H , et al. Multidisciplinary consensus guideline for the diagnosis and management of spontaneous intracranial hypotension. J Neurol Neurosurg Psychiatry. 2023;94(10):835‐843. 37147116 10.1136/jnnp-2023-331166 PMC10511987

[18] Schievink WI , Maya MM , Moser FG . Digital subtraction myelography in the investigation of post‐dural puncture headache in 27 patients: technical note. J Neurosurg Spine. 2017;26(6):760‐764. 28362213 10.3171/2016.11.SPINE16968

[19] Tay ASMS , Maya M , Moser FG , Nuño M , Schievink WI . Computed tomography vs heavily T2‐weighted magnetic resonance myelography for the initial evaluation of patients with spontaneous intracranial hypotension. JAMA Neurol. 2021;78(10):1275‐1276. 34459855 10.1001/jamaneurol.2021.2868 PMC8406204

[20] Barad M , Carroll I , Reina MA , Ansari J , Flood P . Did she have an epidural? The long‐term consequences of postdural puncture headache and the role of unintended dural puncture. Headache. 2021;61(9):1314‐1323. 34570902 10.1111/head.14221

[21] Statement on Post‐Dural Puncture Headache Management [Internet]. [cited 2025 Jan 12]. https://www.asahq.org/standards‐and‐practice‐parameters/statement‐on‐post‐dural‐puncture‐headache‐management

[22] Lee GH , Kim J , Kim HW , Cho JW . Comparisons of clinical characteristics, brain MRI findings, and responses to epidural blood patch between spontaneous intracranial hypotension and post‐dural puncture headache: retrospective study. BMC Neurol. 2021;21(1):253. 34187377 10.1186/s12883-021-02279-5 PMC8243531

[23] Niraj G , Critchley P . Management and outcomes of persistent headache after accidental dural puncture in the obstetric population: a 9‐year prospective audit. Headache. 2023;63(1):71‐78. 36651506 10.1111/head.14447

[24] Kang CH , Madhavan AA , Benson JC , et al. Evaluation of spontaneous intracranial hypotension probabilistic brain MRI scoring systems in normal patients. AJNR Am J Neuroradiol. 2025;46(9):1925‐1930. doi:10.3174/ajnr.A8713 39979026 PMC12453467

[25] Kranz PG , Amrhein TJ , Choudhury KR , Tanpitukpongse TP , Gray L . Time‐dependent changes in dural enhancement associated with spontaneous intracranial hypotension. AJR Am J Roentgenol. 2016;207(6):1283‐1287. 27557149 10.2214/AJR.16.16381

[26] Arevalo‐Rodriguez I , Muñoz L , Godoy‐Casasbuenas N , et al. Needle gauge and tip designs for preventing post‐dural puncture headache (PDPH). Cochrane Database Syst Rev. 2017;4(4):CD010807. 28388808 10.1002/14651858.CD010807.pub2 PMC6478120

[27] Turnbull DK , Shepherd DB . Post‐dural puncture headache: pathogenesis, prevention and treatment. Br J Anaesth. 2003;91(5):718‐729. 14570796 10.1093/bja/aeg231

[28] Banks S , Paech M , Gurrin L . An audit of epidural blood patch after accidental dural puncture with a Tuohy needle in obstetric patients. Int J Obstet Anesth. 2001;10(3):172‐176. 15321606 10.1054/ijoa.2000.0826

[29] Shah LM , McLean LA , Heilbrun ME , Salzman KL . Intracranial hypotension: improved MRI detection with diagnostic intracranial angles. AJR Am J Roentgenol. 2013;200(2):400‐407. 23345364 10.2214/AJR.12.8611

[30] Shankar JJS , Chakraborty S , Lum C . The venous hinge—an objective sign for the diagnosis and follow‐up of treatment in patients with intracranial hypotension syndrome. Neuroradiology. 2009;51(7):453‐456. 19305986 10.1007/s00234-009-0518-7

[31] Johnson DR , Carr CM , Luetmer PH , et al. Diffuse calvarial hyperostosis in patients with spontaneous intracranial hypotension. World Neurosurg. 2021;146:e848‐e853. 33220476 10.1016/j.wneu.2020.11.066

[32] Schievink WI , Maya MM , Tay ASS , et al. Optic nerve sheath MR imaging measurements in patients with orthostatic headaches and normal findings on conventional imaging predict the presence of an underlying CSF‐venous fistula. AJNR Am J Neuroradiol. 2024;45(5):655‐661. doi:10.3174/ajnr.A8165 38485201 PMC11288531

[33] Sakurai K , Kanoto M , Nakagawa M , et al. Dinosaur tail sign: a useful spinal MRI finding indicative of cerebrospinal fluid leakage. Headache. 2017;57(6):917‐925. 28419438 10.1111/head.13075

[34] Roytman M , Ulrich CT , Chazen JL . Post‐dural puncture pseudomeningocele (“arachnoid bleb”): an underrecognized etiology of spontaneous intracranial hypotension symptomatology. Clin Imaging. 2021;80(1):377‐381. 34517304 10.1016/j.clinimag.2021.08.023

[35] Callen AL , Lennarson P , Carroll IR . A causative role for remote dural puncture and resultant arachnoid bleb in new daily persistent headache: a case report. Headache. 2023;63(7):981‐983. 37358488 10.1111/head.14584

