# Supplementary material for: Patients with chronic post‐dural puncture headache do not have typical imaging features of intracranial hypotension: An MRI study using the Bern score
Source: Headache. 2025 Sep 23;66(1):193–201. doi: 10.1111/head.15057 (PMC12849523; doi:10.1111/head.15057)
Supplement: Supplementary file 1 — Table S1: Technical data of used imaging protocols for head and spine for Prisma (3 T)/Avanto (1.5 T). All patients included in the study received 3D T1‐weighted contrast enhanced magnetic resonance imaging of the brain (MPRage). For assessment of epidural lumbar fluid, heavily‐T2‐weighted imaging of the lumbar spine was used, where available (i.e. 3D T2‐weighted SPAIR or 3D T2‐weighted SPACE fat‐sat). Imaging was either done in 1.5 Tesla scanner (Avanto, Siemens, Erlangen, Germany) or 3 Tesla (Prisma, Siemens, Erlangen, Germany). FA, flip angle; MPRAGE, 3D Magnetization Prepared Rapid Gradient Echo; SPACE, sampling perfection with application‐optimized contrasts by using different flip angle evolutions; SPAIR, spectral attenuated inversion recovery; TE, echo time; TR, repetition time. Table S2: Results of the evaluation of the Bern score in 86 chronic postdural puncture headache patients comparing each minor and major criterium between the subgroups after lumbar puncture (LP) versus after peridural anesthesia (PDA). After testing using the chi‐squared test, no significant differences were found for any of the signs between the two subgroups. [file HEAD-66-193-s001.docx]

Online supplementary Table 1: Technical data of used imaging protocols for head and spine for Prisma (3T) / Avanto (1.5T). All patients included in the study received 3D T1-weighted contrast enhanced magnetic resonance imaging of the brain (MPRage). For assessment of epidural lumbar fluid, heavily-T2-weighted imaging of the lumbar spine was used, where available (i.e. 3D T2-weighted SPAIR or 3D T2-weighted SPACE fat-sat). Imaging was either done in 1.5 Tesla scanner (Avanto, Siemens, Erlangen, Germany) or 3 Tesla (Prisma, Siemens, Erlangen, Germany). MPRAGE = 3D Magnetization Prepared Rapid Gradient Echo; SPAIR = spectral attenuated inversion recovery; SPACE = sampling perfection with application-optimized contrasts by using different flip angle evolutions; TR = repetition time; TE = echo time; FA = flip angle


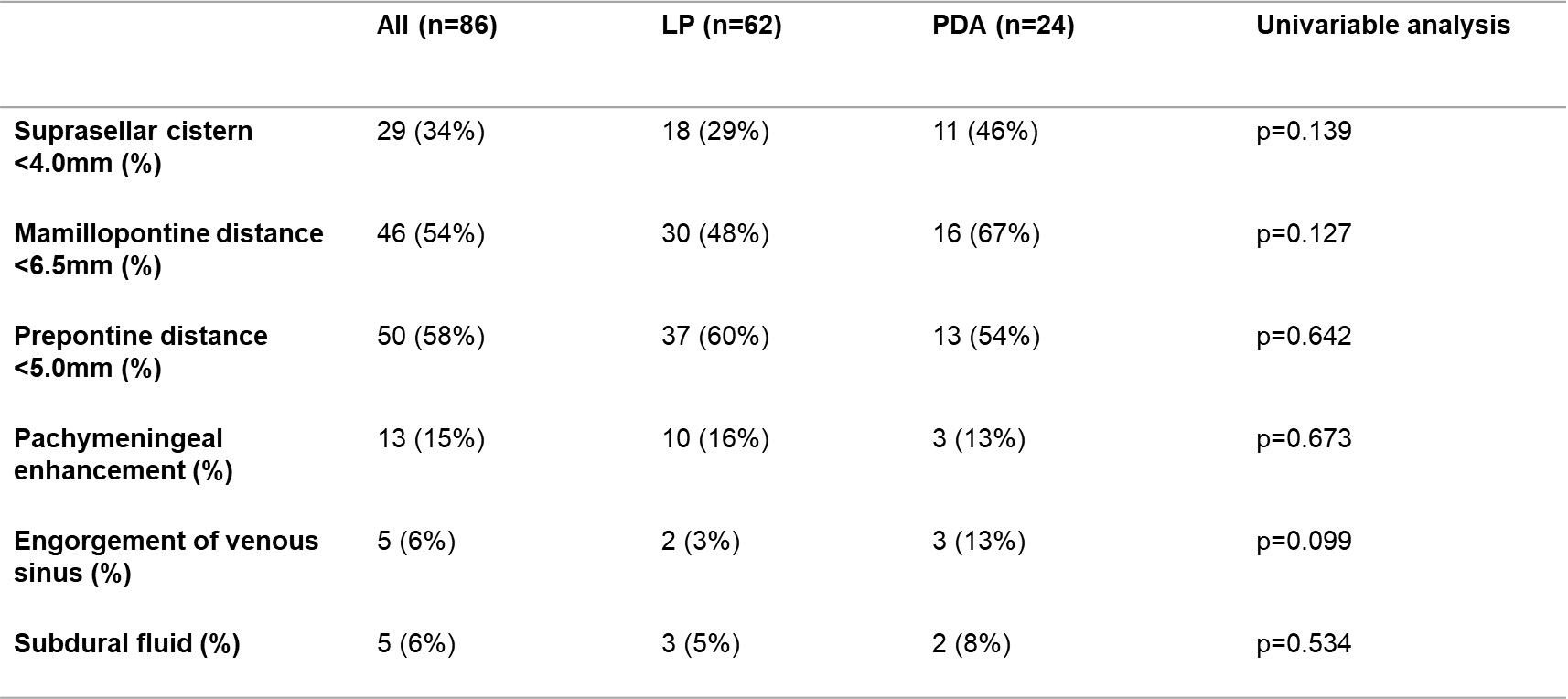


Online supplementary Table 2: Results of the evaluation of the Bern Score in 86 chronic postdural puncture headache patients comparing each minor and major criterium between the subgroups after lumbar puncture (LP) vs. after peridural anesthesia (PDA). After testing using the chi-squared test, no significant differences were found for any of the signs between the two subgroups.
